# 9-(4-Chloro­phen­oxy­carbon­yl)-10-methyl­acridinium trifluoro­methane­sulfonate

**DOI:** 10.1107/S1600536810039541

**Published:** 2010-10-09

**Authors:** Damian Trzybiński, Karol Krzymiński, Jerzy Błażejowski

**Affiliations:** a Faculty of Chemistry, University of Gdańsk, J. Sobieskiego 18, 80-952 Gdańsk, Poland

## Abstract

In the crystal of the title compound, C_21_H_15_ClNO_2_^+^·CF_3_SO_3_^−^, adjacent cations are linked through C—H⋯π and π–π inter­actions [centroid–centroid distance = 3.987 (2) Å], and neighboring cations and anions *via* C—H⋯O and C—F⋯π inter­actions. The acridine ring system and benzene ring are oriented at a dihedral angle of 1.0 (1)° while the carboxyl group is twisted at an angle of 85.0 (1)° relative to the acridine skeleton. The mean planes of adjacent acridine units are either parallel or inclined at an angle of 78.2 (1)° in the crystal structure.

## Related literature

For background to the chemiluminogenic properties of 9-phen­oxy­carbonyl-10-methyl­acridinium trifluoro­methane­sulfonates, see: Brown *et al.* (2009 ▶); King *et al.* (2007 ▶); Rak *et al.* (1999 ▶); Roda *et al.* (2003 ▶); Zomer & Jacquemijns (2001 ▶). For related structures, see: Sikorski *et al.* (2005 ▶); Trzybiński *et al.* (2010 ▶). For inter­molecular inter­actions, see: Dorn *et al.* (2005 ▶); Hunter *et al.* (2001 ▶); Novoa *et al.* (2006 ▶); Takahashi *et al.* (2001 ▶). For the synthesis, see: Sato (1996 ▶); Trzybiński *et al.* (2010 ▶).
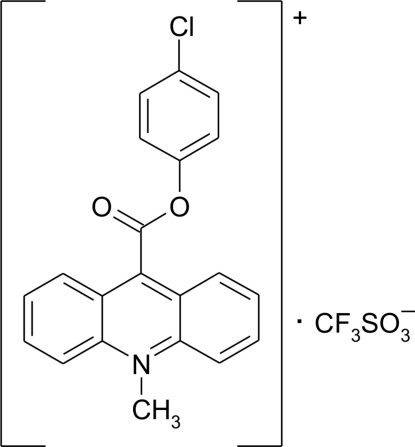


## Experimental

### 

#### Crystal data

C_21_H_15_ClNO_2_^+^·CF_3_SO_3_^−^
*M* *_r_* = 497.87
Monoclinic, 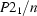

*a* = 13.3025 (11) Å
*b* = 8.6750 (9) Å
*c* = 19.6191 (18) Å
β = 106.577 (10)°
*V* = 2169.9 (4) Å^3^
*Z* = 4
Mo *K*α radiation
μ = 0.33 mm^−1^
*T* = 295 K
0.35 × 0.28 × 0.06 mm

#### Data collection

Oxford Diffraction Gemini R Ultra Ruby CCD diffractometer
11162 measured reflections
3777 independent reflections
2679 reflections with *I* > 2σ(*I*)
*R*_int_ = 0.031

#### Refinement

*R*[*F* ^2^ > 2σ(*F* ^2^)] = 0.050
*wR*(*F* ^2^) = 0.112
*S* = 1.08
3777 reflections
299 parameters
H-atom parameters constrained
Δρ_max_ = 0.24 e Å^−3^
Δρ_min_ = −0.29 e Å^−3^

### 

Data collection: *CrysAlis CCD* (Oxford Diffraction, 2008 ▶); cell refinement: *CrysAlis RED* (Oxford Diffraction, 2008 ▶); data reduction: *CrysAlis RED*; program(s) used to solve structure: *SHELXS97* (Sheldrick, 2008 ▶); program(s) used to refine structure: *SHELXL97* (Sheldrick, 2008 ▶); molecular graphics: *ORTEP-3* (Farrugia, 1997 ▶); software used to prepare material for publication: *SHELXL97* and *PLATON* (Spek, 2009 ▶).

## Supplementary Material

Crystal structure: contains datablocks global, I. DOI: 10.1107/S1600536810039541/xu5039sup1.cif

Structure factors: contains datablocks I. DOI: 10.1107/S1600536810039541/xu5039Isup2.hkl

Additional supplementary materials:  crystallographic information; 3D view; checkCIF report

## Figures and Tables

**Table 1:** Hydrogen-bond geometry (Å, °) *Cg*4 is the centroid of the C18–C23 ring.

*D*—H⋯*A*	*D*—H	H⋯*A*	*D*⋯*A*	*D*—H⋯*A*
C3—H3⋯O28^i^	0.93	2.59	3.328 (5)	136
C4—H4⋯O28	0.93	2.45	3.370 (4)	171
C5—H5⋯O27^ii^	0.93	2.39	3.258 (4)	154
C6—H6⋯O29^ii^	0.93	2.54	3.304 (5)	140
C8—H8⋯O29^iii^	0.93	2.59	3.332 (4)	137
C19—H19⋯O29^iii^	0.93	2.44	3.349 (4)	165
C25—H25*C*⋯O27	0.96	2.51	3.387 (4)	152
C25—H25*B*⋯*Cg*4^iv^	0.96	2.65	3.519 (4)	151

Symmetry codes: (i) 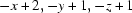; (ii) 
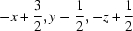
; (iii) 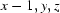; (iv) 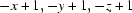.

**Table 2:** C–F⋯π inter­actions (Å,°) *Cg*1 and *Cg*3 are the centroids of the C9/N10/C11–C14 and C5–C8/C13/C14 rings, respectively.

*X*	*I*	*J*	*I*⋯*J*	*X*⋯*J*	*X*–*I*⋯*J*
C30	F31	*Cg*1^ii^	3.570 (3)	3.916 (4)	94.4 (2)
C30	F32	*Cg*1^ii^	3.337 (3)	3.916 (4)	105.8 (2)
C30	F33	*Cg*3^ii^	3.387 (3)	4.073 (4)	111.9 (2)

Symmetry code: (ii) 
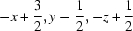
.

**Fig. 1.**
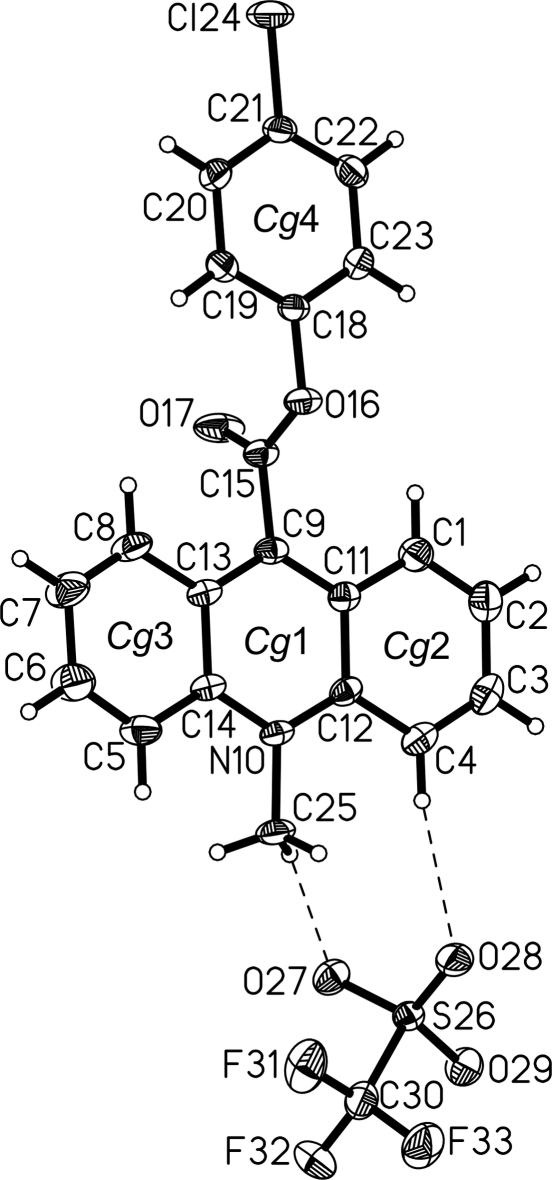
The molecular structure of the title compound showing the atom-labeling scheme. Displacement ellipsoids are drawn at the 25% probability level and H atoms are shown as small spheres of arbitrary radius. Cg1, Cg2, Cg3 and Cg4 denote the ring centroids. The C–H···O interactions are represented by dashed lines.

**Fig. 2.**
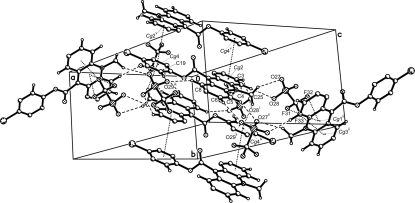
The arrangement of the ions in the crystal structure. The C–H···O interactions are represented by dashed lines, the C–H···π, C–F···π, and π–π contacts by dotted lines. H atoms not involved in interactions have been omitted. [Symmetry codes: (i) –x + 2, –y + 1, –z + 1; (ii) –x + 3/2, y – 1/2, –z + 1/2; (iii) x – 1, y, z; (iv) –x + 1, –y + 1, –z + 1; (v) –x + 1, –y + 2, –z + 1.]

## supplementary crystallographic information

### Comment

The long-known chemiluminescence of 9-(phenoxycarbonyl)-10-methylacridinium salts has been used as chemiluminescent indicators and labels that are widely applied in assays of biologically and environmentally important entities such as antigens, antibodies, enzymes or DNA fragments (Zomer & Jacquemijns, 2001; Roda *et al.*, 2003; King *et al.*, 2007; Brown *et al.*, 2009). The cations of these salts are oxidized by H_2_O_2_ in alkaline media, a reaction that is accompanied by the removal of the phenoxycarbonyl fragment and the conversion of the remaining part of the molecules to electronically excited, light-emitting 10-methyl-9-acridinone (Rak *et al.*, 1999). The efficiency of chemiluminescence – crucial for analytical applications – is affected by the constitution of the phenyl fragment (Zomer & Jacquemijns, 2001). In continuing our investigations on the latter aspect, we synthesized 9-(4-chlorophenoxycarbonyl)-10-methylacridinium trifluoromethanesulfonate, whose crystal structure is presented here.

In the cation of the title compound (Fig. 1), the bond lengths and angles characterizing the geometry of the acridinium moiety are typical of acridine-based derivatives (Sikorski *et al.*, 2005; Trzybiński *et al.*, 2010). With respective average deviations from planarity of 0.0412 (3) Å and 0.0034 (3) Å, the acridine and benzene ring systems are almost parallel (are oriented at a dihedral angle of 1.0 (1)°). The carboxyl group is twisted at an angle of 85.0 (1)° relative to the acridine skeleton. The mean planes of the adjacent acridine moieties are parallel (remain at an angle 0.0 (1)°) or inclined at an angle of 78.2 (1)° in the crystal lattice.

In the crystal structure, the adjacent cations are linked by C–H···π (Table 1, Fig. 2) and π-π (Table 3, Fig.2) contacts, and the cations and neighboring anions via C–H···O (Table 1, Figs. 1 and 2) and C–F···π (Table 2, Fig. 2) interactions. The C–H···O interactions are of the hydrogen bond type (Novoa *et al.* 2006). The C–H···π (Takahashi *et al.*, 2001), C–F···π (Dorn *et al.*, 2005) and π–π (Hunter *et al.*, 2001) interactions should be of an attractive nature. The crystal structure is stabilized by a network of these short-range specific interactions and by long-range electrostatic interactions between ions.

### Experimental

4-Chlorophenylacridine-9-carboxylate was synthesized by esterification of 9-(chlorocarbonyl)acridine (obtained by treating acridine-9-carboxylic acid with a tenfold molar excess of thionyl chloride) with 4-chlorophenol in anhydrous dichloromethane in the presence of *N*,*N*-diethylethanamine and a catalytic amount of *N*,*N*-dimethyl-4-pyridinamine (room temperature, 15h) (Sato, 1996). The product was purified chromatographically (SiO_2_, cyclohexane/ethyl acetate, 1/1 v/v) and subsequently quaternarized with a fivefold molar excess of methyl trifluoromethanesulfonate dissolved in anhydrous dichloromethane. The crude 9-(4-chlorophenoxycarbonyl)-10-methylacridinium trifluoromethanesulfonate was dissolved in a small amount of ethanol, filtered and precipitated with a 20 v/v excess of diethyl ether. Yellow crystals suitable for X-ray investigations were grown from absolute ethanol solution (m.p. 488–489 K).

### Refinement

H atoms were positioned geometrically, with C—H = 0.93 Å and 0.96 Å for the aromatic and methyl H atoms, respectively, and constrained to ride on their parent atoms with *U*_iso_(H) = *xU*_eq_(C), where *x* = 1.2 for the aromatic and *x* = 1.5 for the methyl H atoms.

### Crystal data

**Table:**

C_21_H_15_ClNO_2_^+^·CF_3_SO_3_^−^	*F*(000) = 1016
*M**_r_* = 497.87	*D*_x_ = 1.524 Mg m^−^^3^
Monoclinic, *P*2_1_/*n*	Mo *K*α radiation, λ = 0.71073 Å
Hall symbol: -P 2yn	Cell parameters from 1665 reflections
*a* = 13.3025 (11) Å	θ = 3.0–29.1°
*b* = 8.6750 (9) Å	µ = 0.33 mm^−^^1^
*c* = 19.6191 (18) Å	*T* = 295 K
β = 106.577 (10)°	Plate, yellow
*V* = 2169.9 (4) Å^3^	0.35 × 0.28 × 0.06 mm
*Z* = 4	

### Data collection

**Table:**

Oxford Diffraction Gemini R Ultra Ruby CCD diffractometer	2679 reflections with *I* > 2σ(*I*)
Radiation source: Enhanced (Mo) X-ray Source	*R*_int_ = 0.031
graphite	θ_max_ = 25.1°, θ_min_ = 3.2°
Detector resolution: 10.4002 pixels mm^-1^	*h* = −15→15
ω scans	*k* = −10→8
11162 measured reflections	*l* = −23→23
3777 independent reflections	

### Refinement

**Table:**

Refinement on *F*^2^	Primary atom site location: structure-invariant direct methods
Least-squares matrix: full	Secondary atom site location: difference Fourier map
*R*[*F*^2^ > 2σ(*F*^2^)] = 0.050	Hydrogen site location: inferred from neighbouring sites
*wR*(*F*^2^) = 0.112	H-atom parameters constrained
*S* = 1.08	*w* = 1/[σ^2^(*F*_o_^2^) + (0.0386*P*)^2^ + 1.4339*P*] where *P* = (*F*_o_^2^ + 2*F*_c_^2^)/3
3777 reflections	(Δ/σ)_max_ < 0.001
299 parameters	Δρ_max_ = 0.24 e Å^−^^3^
0 restraints	Δρ_min_ = −0.29 e Å^−^^3^

### Special details

**Table:**

Geometry. All esds (except the esd in the dihedral angle between two l.s. planes) are estimated using the full covariance matrix. The cell esds are taken into account individually in the estimation of esds in distances, angles and torsion angles; correlations between esds in cell parameters are only used when they are defined by crystal symmetry. An approximate (isotropic) treatment of cell esds is used for estimating esds involving l.s. planes.

### Fractional atomic coordinates and isotropic or equivalent isotropic displacement parameters (Å^2^)

**Table:**

	*x*	*y*	*z*	*U*_iso_*/*U*_eq_	
C1	0.6412 (3)	0.7757 (4)	0.51159 (16)	0.0551 (8)	
H1	0.5968	0.8280	0.5327	0.066*	
C2	0.7459 (3)	0.7893 (4)	0.53941 (18)	0.0655 (9)	
H2	0.7736	0.8503	0.5794	0.079*	
C3	0.8127 (3)	0.7105 (4)	0.50748 (19)	0.0642 (9)	
H3	0.8848	0.7193	0.5274	0.077*	
C4	0.7761 (2)	0.6220 (4)	0.44874 (17)	0.0545 (8)	
H4	0.8226	0.5731	0.4284	0.065*	
C5	0.4792 (3)	0.3888 (4)	0.27537 (17)	0.0610 (9)	
H5	0.5241	0.3355	0.2551	0.073*	
C6	0.3742 (3)	0.3699 (5)	0.2497 (2)	0.0827 (12)	
H6	0.3480	0.3034	0.2116	0.099*	
C7	0.3035 (3)	0.4472 (5)	0.2787 (2)	0.0811 (12)	
H7	0.2316	0.4327	0.2597	0.097*	
C8	0.3403 (2)	0.5423 (4)	0.33414 (19)	0.0610 (9)	
H8	0.2933	0.5927	0.3536	0.073*	
C9	0.4901 (2)	0.6658 (3)	0.42070 (14)	0.0407 (7)	
N10	0.62580 (17)	0.5155 (3)	0.35967 (12)	0.0408 (5)	
C11	0.5976 (2)	0.6831 (3)	0.45086 (14)	0.0408 (7)	
C12	0.6666 (2)	0.6045 (3)	0.41855 (15)	0.0416 (7)	
C13	0.4494 (2)	0.5676 (3)	0.36378 (15)	0.0426 (7)	
C14	0.5202 (2)	0.4893 (3)	0.33286 (14)	0.0421 (7)	
C15	0.4160 (2)	0.7658 (3)	0.44662 (15)	0.0451 (7)	
O16	0.39162 (15)	0.7040 (2)	0.50208 (10)	0.0497 (5)	
O17	0.3831 (2)	0.8846 (3)	0.41934 (13)	0.0785 (8)	
C18	0.3180 (2)	0.7867 (3)	0.52827 (14)	0.0410 (7)	
C19	0.2135 (2)	0.7596 (4)	0.49788 (15)	0.0503 (8)	
H19	0.1910	0.6928	0.4594	0.060*	
C20	0.1421 (2)	0.8339 (4)	0.52579 (16)	0.0566 (8)	
H20	0.0706	0.8176	0.5063	0.068*	
C21	0.1776 (3)	0.9314 (4)	0.58220 (15)	0.0535 (8)	
C22	0.2823 (3)	0.9565 (4)	0.61275 (15)	0.0558 (8)	
H22	0.3049	1.0220	0.6516	0.067*	
C23	0.3544 (2)	0.8829 (3)	0.58496 (15)	0.0484 (7)	
H23	0.4259	0.8987	0.6045	0.058*	
Cl24	0.08737 (9)	1.02661 (14)	0.61618 (5)	0.0951 (4)	
C25	0.6984 (2)	0.4487 (4)	0.32249 (17)	0.0568 (8)	
H25A	0.6590	0.4126	0.2763	0.085*	
H25B	0.7360	0.3641	0.3497	0.085*	
H25C	0.7473	0.5262	0.3173	0.085*	
S26	0.99146 (6)	0.51449 (9)	0.33370 (4)	0.0456 (2)	
O27	0.91136 (17)	0.6102 (2)	0.28913 (11)	0.0578 (6)	
O28	0.96649 (17)	0.4501 (3)	0.39381 (11)	0.0709 (7)	
O29	1.09667 (17)	0.5708 (3)	0.34719 (12)	0.0704 (7)	
C30	0.9908 (3)	0.3493 (4)	0.27794 (18)	0.0641 (9)	
F31	0.89748 (18)	0.2801 (2)	0.25860 (13)	0.0971 (7)	
F32	1.01300 (19)	0.3885 (3)	0.21805 (11)	0.0974 (8)	
F33	1.06074 (19)	0.2433 (3)	0.30961 (12)	0.0989 (8)	

### Atomic displacement parameters (Å^2^)

**Table:**

	*U*^11^	*U*^22^	*U*^33^	*U*^12^	*U*^13^	*U*^23^
C1	0.056 (2)	0.0547 (19)	0.0543 (19)	0.0055 (16)	0.0160 (16)	0.0022 (16)
C2	0.064 (2)	0.065 (2)	0.059 (2)	−0.0006 (19)	0.0042 (18)	−0.0015 (17)
C3	0.0434 (19)	0.072 (2)	0.071 (2)	0.0002 (18)	0.0048 (17)	0.0125 (19)
C4	0.0419 (19)	0.0549 (19)	0.069 (2)	0.0081 (15)	0.0189 (16)	0.0107 (17)
C5	0.057 (2)	0.058 (2)	0.076 (2)	−0.0016 (17)	0.0320 (18)	−0.0180 (18)
C6	0.063 (3)	0.088 (3)	0.098 (3)	−0.015 (2)	0.024 (2)	−0.044 (2)
C7	0.043 (2)	0.094 (3)	0.108 (3)	−0.013 (2)	0.024 (2)	−0.034 (2)
C8	0.0386 (18)	0.065 (2)	0.086 (2)	0.0017 (16)	0.0285 (17)	−0.0115 (19)
C9	0.0458 (18)	0.0348 (15)	0.0479 (16)	0.0071 (13)	0.0235 (14)	0.0073 (13)
N10	0.0377 (13)	0.0403 (13)	0.0514 (13)	0.0090 (11)	0.0240 (11)	0.0084 (11)
C11	0.0454 (18)	0.0338 (15)	0.0455 (16)	0.0060 (13)	0.0167 (14)	0.0081 (12)
C12	0.0377 (17)	0.0388 (16)	0.0512 (17)	0.0065 (13)	0.0172 (13)	0.0121 (14)
C13	0.0396 (17)	0.0387 (15)	0.0554 (17)	0.0060 (13)	0.0227 (14)	0.0039 (13)
C14	0.0396 (17)	0.0370 (15)	0.0560 (17)	0.0045 (13)	0.0237 (14)	0.0027 (13)
C15	0.0490 (18)	0.0407 (17)	0.0517 (17)	0.0095 (14)	0.0239 (15)	0.0049 (14)
O16	0.0550 (13)	0.0479 (12)	0.0560 (12)	0.0168 (10)	0.0316 (10)	0.0107 (9)
O17	0.110 (2)	0.0622 (15)	0.0876 (17)	0.0457 (15)	0.0674 (15)	0.0311 (13)
C18	0.0443 (18)	0.0428 (16)	0.0401 (15)	0.0089 (13)	0.0188 (14)	0.0044 (13)
C19	0.053 (2)	0.0585 (19)	0.0414 (16)	−0.0012 (15)	0.0157 (15)	−0.0035 (14)
C20	0.0387 (18)	0.081 (2)	0.0514 (18)	0.0058 (16)	0.0144 (15)	0.0060 (17)
C21	0.057 (2)	0.068 (2)	0.0418 (16)	0.0242 (17)	0.0241 (15)	0.0113 (15)
C22	0.068 (2)	0.057 (2)	0.0411 (16)	0.0115 (17)	0.0140 (16)	−0.0056 (14)
C23	0.0401 (17)	0.0562 (19)	0.0464 (16)	0.0050 (15)	0.0081 (14)	0.0026 (15)
Cl24	0.0976 (8)	0.1281 (9)	0.0771 (6)	0.0595 (7)	0.0531 (6)	0.0170 (6)
C25	0.0459 (19)	0.069 (2)	0.066 (2)	0.0127 (16)	0.0333 (16)	0.0015 (16)
S26	0.0369 (4)	0.0554 (5)	0.0466 (4)	−0.0024 (4)	0.0151 (3)	−0.0039 (4)
O27	0.0521 (13)	0.0545 (13)	0.0673 (14)	0.0069 (11)	0.0176 (11)	0.0104 (11)
O28	0.0590 (15)	0.1053 (19)	0.0566 (13)	0.0177 (13)	0.0296 (11)	0.0210 (13)
O29	0.0442 (13)	0.0904 (17)	0.0758 (15)	−0.0218 (12)	0.0157 (11)	−0.0182 (13)
C30	0.053 (2)	0.069 (2)	0.063 (2)	0.0070 (19)	0.0048 (17)	−0.0033 (18)
F31	0.0796 (16)	0.0704 (14)	0.1195 (18)	−0.0186 (12)	−0.0069 (14)	−0.0203 (13)
F32	0.1092 (19)	0.130 (2)	0.0568 (12)	0.0208 (15)	0.0297 (12)	−0.0186 (13)
F33	0.0931 (17)	0.0805 (15)	0.1065 (17)	0.0384 (13)	0.0016 (14)	−0.0116 (13)

### Geometric parameters (Å, °)

**Table:**

C1—C2	1.349 (4)	C13—C14	1.428 (4)
C1—C11	1.417 (4)	C15—O17	1.186 (3)
C1—H1	0.9300	C15—O16	1.334 (3)
C2—C3	1.402 (5)	O16—C18	1.423 (3)
C2—H2	0.9300	C18—C23	1.364 (4)
C3—C4	1.354 (5)	C18—C19	1.367 (4)
C3—H3	0.9300	C19—C20	1.384 (4)
C4—C12	1.415 (4)	C19—H19	0.9300
C4—H4	0.9300	C20—C21	1.366 (4)
C5—C6	1.353 (5)	C20—H20	0.9300
C5—C14	1.407 (4)	C21—C22	1.368 (4)
C5—H5	0.9300	C21—Cl24	1.739 (3)
C6—C7	1.401 (5)	C22—C23	1.386 (4)
C6—H6	0.9300	C22—H22	0.9300
C7—C8	1.341 (5)	C23—H23	0.9300
C7—H7	0.9300	C25—H25A	0.9600
C8—C13	1.418 (4)	C25—H25B	0.9600
C8—H8	0.9300	C25—H25C	0.9600
C9—C13	1.386 (4)	S26—O28	1.427 (2)
C9—C11	1.391 (4)	S26—O29	1.434 (2)
C9—C15	1.506 (4)	S26—O27	1.434 (2)
N10—C12	1.367 (4)	S26—C30	1.801 (4)
N10—C14	1.372 (3)	C30—F33	1.330 (4)
N10—C25	1.485 (3)	C30—F31	1.333 (4)
C11—C12	1.429 (4)	C30—F32	1.335 (4)
			
C2—C1—C11	121.2 (3)	C5—C14—C13	118.9 (3)
C2—C1—H1	119.4	O17—C15—O16	125.0 (3)
C11—C1—H1	119.4	O17—C15—C9	122.7 (3)
C1—C2—C3	119.3 (3)	O16—C15—C9	112.2 (2)
C1—C2—H2	120.4	C15—O16—C18	116.4 (2)
C3—C2—H2	120.4	C23—C18—C19	122.7 (3)
C4—C3—C2	122.5 (3)	C23—C18—O16	118.8 (3)
C4—C3—H3	118.8	C19—C18—O16	118.3 (2)
C2—C3—H3	118.8	C18—C19—C20	118.3 (3)
C3—C4—C12	119.6 (3)	C18—C19—H19	120.8
C3—C4—H4	120.2	C20—C19—H19	120.8
C12—C4—H4	120.2	C21—C20—C19	119.5 (3)
C6—C5—C14	119.7 (3)	C21—C20—H20	120.3
C6—C5—H5	120.1	C19—C20—H20	120.3
C14—C5—H5	120.1	C20—C21—C22	121.8 (3)
C5—C6—C7	122.2 (3)	C20—C21—Cl24	119.2 (3)
C5—C6—H6	118.9	C22—C21—Cl24	119.0 (2)
C7—C6—H6	118.9	C21—C22—C23	119.1 (3)
C8—C7—C6	119.4 (3)	C21—C22—H22	120.4
C8—C7—H7	120.3	C23—C22—H22	120.4
C6—C7—H7	120.3	C18—C23—C22	118.6 (3)
C7—C8—C13	121.5 (3)	C18—C23—H23	120.7
C7—C8—H8	119.3	C22—C23—H23	120.7
C13—C8—H8	119.3	N10—C25—H25A	109.5
C13—C9—C11	121.7 (2)	N10—C25—H25B	109.5
C13—C9—C15	118.9 (3)	H25A—C25—H25B	109.5
C11—C9—C15	119.1 (3)	N10—C25—H25C	109.5
C12—N10—C14	122.3 (2)	H25A—C25—H25C	109.5
C12—N10—C25	118.6 (2)	H25B—C25—H25C	109.5
C14—N10—C25	119.1 (2)	O28—S26—O29	115.20 (14)
C9—C11—C1	122.9 (3)	O28—S26—O27	115.00 (13)
C9—C11—C12	118.2 (3)	O29—S26—O27	115.57 (14)
C1—C11—C12	118.9 (3)	O28—S26—C30	103.23 (17)
N10—C12—C4	121.8 (3)	O29—S26—C30	102.62 (16)
N10—C12—C11	119.6 (3)	O27—S26—C30	102.48 (14)
C4—C12—C11	118.6 (3)	F33—C30—F31	107.0 (3)
C9—C13—C8	122.9 (3)	F33—C30—F32	106.6 (3)
C9—C13—C14	118.8 (3)	F31—C30—F32	106.6 (3)
C8—C13—C14	118.3 (3)	F33—C30—S26	112.6 (2)
N10—C14—C5	122.0 (2)	F31—C30—S26	112.0 (2)
N10—C14—C13	119.1 (2)	F32—C30—S26	111.6 (3)
			
C11—C1—C2—C3	0.1 (5)	C6—C5—C14—C13	1.1 (5)
C1—C2—C3—C4	0.9 (5)	C9—C13—C14—N10	−1.0 (4)
C2—C3—C4—C12	−1.3 (5)	C8—C13—C14—N10	178.3 (3)
C14—C5—C6—C7	−0.3 (6)	C9—C13—C14—C5	179.6 (3)
C5—C6—C7—C8	−0.6 (7)	C8—C13—C14—C5	−1.1 (4)
C6—C7—C8—C13	0.6 (6)	C13—C9—C15—O17	−82.2 (4)
C13—C9—C11—C1	−176.2 (3)	C11—C9—C15—O17	92.9 (4)
C15—C9—C11—C1	8.8 (4)	C13—C9—C15—O16	96.9 (3)
C13—C9—C11—C12	4.3 (4)	C11—C9—C15—O16	−88.0 (3)
C15—C9—C11—C12	−170.7 (2)	O17—C15—O16—C18	1.9 (4)
C2—C1—C11—C9	179.9 (3)	C9—C15—O16—C18	−177.1 (2)
C2—C1—C11—C12	−0.6 (4)	C15—O16—C18—C23	−96.8 (3)
C14—N10—C12—C4	175.5 (2)	C15—O16—C18—C19	86.8 (3)
C25—N10—C12—C4	−6.3 (4)	C23—C18—C19—C20	0.4 (4)
C14—N10—C12—C11	−4.7 (4)	O16—C18—C19—C20	176.7 (3)
C25—N10—C12—C11	173.5 (2)	C18—C19—C20—C21	0.1 (5)
C3—C4—C12—N10	−179.4 (3)	C19—C20—C21—C22	−0.9 (5)
C3—C4—C12—C11	0.8 (4)	C19—C20—C21—Cl24	179.1 (2)
C9—C11—C12—N10	−0.1 (4)	C20—C21—C22—C23	1.1 (5)
C1—C11—C12—N10	−179.6 (2)	Cl24—C21—C22—C23	−178.9 (2)
C9—C11—C12—C4	179.7 (2)	C19—C18—C23—C22	−0.2 (4)
C1—C11—C12—C4	0.2 (4)	O16—C18—C23—C22	−176.4 (2)
C11—C9—C13—C8	177.0 (3)	C21—C22—C23—C18	−0.6 (4)
C15—C9—C13—C8	−8.0 (4)	O28—S26—C30—F33	−60.1 (3)
C11—C9—C13—C14	−3.8 (4)	O29—S26—C30—F33	60.0 (3)
C15—C9—C13—C14	171.2 (2)	O27—S26—C30—F33	−179.9 (3)
C7—C8—C13—C9	179.5 (3)	O28—S26—C30—F31	60.5 (3)
C7—C8—C13—C14	0.3 (5)	O29—S26—C30—F31	−179.4 (2)
C12—N10—C14—C5	−175.4 (3)	O27—S26—C30—F31	−59.2 (3)
C25—N10—C14—C5	6.4 (4)	O28—S26—C30—F32	180.0 (2)
C12—N10—C14—C13	5.2 (4)	O29—S26—C30—F32	−60.0 (3)
C25—N10—C14—C13	−173.0 (2)	O27—S26—C30—F32	60.2 (3)
C6—C5—C14—N10	−178.3 (3)		

### Hydrogen-bond geometry (Å, °)

**Table:**

Cg4 is the centroid of the C18–C23 ring.

**Table:**

*D*—H···*A*	*D*—H	H···*A*	*D*···*A*	*D*—H···*A*
C3—H3···O28^i^	0.93	2.59	3.328 (5)	136
C4—H4···O28	0.93	2.45	3.370 (4)	171
C5—H5···O27^ii^	0.93	2.39	3.258 (4)	154
C6—H6···O29^ii^	0.93	2.54	3.304 (5)	140
C8—H8···O29^iii^	0.93	2.59	3.332 (4)	137
C19—H19···O29^iii^	0.93	2.44	3.349 (4)	165
C25—H25C···O27	0.96	2.51	3.387 (4)	152
C25—H25B···Cg4^iv^	0.96	2.65	3.519 (4)	151

Symmetry codes:  (i) −*x*+2, −*y*+1, −*z*+1; (ii) −*x*+3/2, *y*−1/2, −*z*+1/2; (iii) *x*−1, *y*, *z*; (iv) −*x*+1, −*y*+1, −*z*+1.

### Table 2 C–F···π interactions (Å,°).

*Cg*1 and *Cg*3 are the centroids of the C9/N10/C11–C14 and C5–C8/C13/C14 rings, respectively.

**Table:**

*X*	*I*	*J*	*I*···*J*	*X*···*J*	*X*–*I*···*J*
C30	F31	*Cg*1^ii^	3.570 (3)	3.916 (4)	94.4 (2)
C30	F32	*Cg*1^ii^	3.337 (3)	3.916 (4)	105.8 (2)
C30	F33	*Cg*3^ii^	3.387 (3)	4.073 (4)	111.9 (2)

Symmetry code: (ii) -*x* + 3/2, *y* - 1/2, -*z* + 1/2.

### Table 3 π–π interactions (Å,°).

**Table:**

*I*	*J*	*CgI*···*CgJ*	Dihedral angle	*CgI*_Perp	Cg*I*_Offset
2	4^v^	3.987 (2)	2.96 (15)	3.477 (2)	1.951 (2)

Symmetry code: (v) –*x* + 1, –*y* + 2, –*z* + 1.Notes: *Cg*2 and *Cg*4 are the centroids of the C1–C4/C11/C12 and C18–C23 rings, respectively. Cg*I*···Cg*J* is the distance between ring centroids. The dihedral angle is that between the planes of the rings *I* and *J*. *CgI*_Perp is the perpendicular distance of *CgI* from ring *J*. Cg*I*_Offset is the distance between *CgI* and perpendicular projection of *CgJ* on ring *I*.
